# 3D Co-culture of hiPSC-Derived Cardiomyocytes With Cardiac Fibroblasts Improves Tissue-Like Features of Cardiac Spheroids

**DOI:** 10.3389/fmolb.2020.00014

**Published:** 2020-02-14

**Authors:** Philippe Beauchamp, Christopher B. Jackson, Lijo Cherian Ozhathil, Irina Agarkova, Cristi L. Galindo, Douglas B. Sawyer, Thomas M. Suter, Christian Zuppinger

**Affiliations:** ^1^Cardiology Department, DBMR MEM C812, Bern University Hospital, Bern, Switzerland; ^2^Stem Cells and Metabolism Research Program, Faculty of Medicine, University of Helsinki, Helsinki, Finland; ^3^IKELOS GmbH, Bern, Switzerland; ^4^Department of Biomedical Sciences, University of Copenhagen, Copenhagen, Denmark; ^5^InSphero AG, Schlieren, Switzerland; ^6^Division of Cardiovascular Medicine, Vanderbilt University Medical School, Nashville, TN, United States; ^7^Department of Cell Biology and Molecular Biology, Rutgers New Jersey Medical School, Newark, NJ, United States; ^8^Department of Cardiac Services, Maine Medical Center, Scarborough, ME, United States

**Keywords:** 3D-culture, induced pluripotent stem cells, cardiomyocyte, fibroblast, myofibroblast, microtissue, scaffold-free, co-culture

## Abstract

**Purpose:** Both cardiomyocytes and cardiac fibroblasts (CF) play essential roles in cardiac development, function, and remodeling. Properties of 3D co-cultures are incompletely understood. Hence, 3D co-culture of cardiomyocytes and CF was characterized, and selected features compared with single-type and 2D culture conditions.

**Methods:** Human cardiomyocytes derived from induced-pluripotent stem cells (hiPSC-CMs) were obtained from Cellular Dynamics or Ncardia, and primary human cardiac fibroblasts from ScienCell. Cardiac spheroids were investigated using cryosections and whole-mount confocal microscopy, video motion analysis, scanning-, and transmission-electron microscopy (SEM, TEM), action potential recording, and quantitative PCR (qPCR).

**Results:** Spheroids formed in hanging drops or in non-adhesive wells showed spontaneous contractions for at least 1 month with frequent media changes. SEM of mechanically opened spheroids revealed a dense inner structure and no signs of blebbing. TEM of co-culture spheroids at 1 month showed myofibrils, intercalated disc-like structures and mitochondria. Ultrastructural features were comparable to fetal human myocardium. We then assessed immunostained 2D cultures, cryosections of spheroids, and whole-mount preparations by confocal microscopy. CF in co-culture spheroids assumed a small size and shape similar to the situation in ventricular tissue. Spheroids made only of CF and cultured for 3 weeks showed no stress fibers and strongly reduced amounts of alpha smooth muscle actin compared to early spheroids and 2D cultures as shown by confocal microscopy, western blotting, and qPCR. The addition of CF to cardiac spheroids did not lead to arrhythmogenic effects as measured by sharp-electrode electrophysiology. Video motion analysis showed a faster spontaneous contraction rate in co-culture spheroids compared to pure hiPSC-CMs, but similar contraction amplitudes and kinetics. Spontaneous contraction rates were not dependent on spheroid size. Applying increasing pacing frequencies resulted in decreasing contraction amplitudes without positive staircase effect. Gene expression analysis of selected cytoskeleton and myofibrillar proteins showed more tissue-like expression patterns in co-culture spheroids than with cardiomyocytes alone or in 2D culture.

**Conclusion:** We demonstrate that the use of 3D co-culture of hiPSC-CMs and CF is superior over 2D culture conditions for co-culture models and more closely mimicking the native state of the myocardium with relevance to drug development as well as for personalized medicine.

## Introduction

Three-dimensional (3D) culture is an alternative to classic cell culture using flat surfaces in flasks and dishes and can help bridge the gap between 2D culture and native tissue (Abbott, 2003; Bissell, 2017; Verjans et al., 2018). We have previously investigated cardiac spheroids made of cardiomyocytes only, which provide a simplified 3D model of the myocardium (Beauchamp et al., 2015). A number of studies have made use of cardiac scaffold-free microtissues, also called spheroids, for drug testing and toxicology, using a mix of several cell types such as rodent or human, primary- or hiPSC-derived cardiomyocytes, fibroblasts, stem cells, and endothelial cells (Garzoni et al., 2009). The influence of non-myocytes has been neglected in simple cardiomyocyte *in vitro* models (Archer et al., 2018). The outcome of co-culturing several cardiac cell types in 3D cultures is incompletely understood and adds complexity and practical challenges. Although adding multiple cell types might mimic the composition of the original tissue better, the role of the endothelial component is not very well-defined in spheroid models as the cells are less well-organized than *in vivo*. To address these issues, we decided to study two cell types, hiPSC-derived human cardiomyocytes (hiPSC-CMs) and primary human cardiac fibroblasts (CF), comparing single-type 3D- and 2D-culture conditions. Since CF produce native extracellular matrix (ECM) proteins and show bidirectional signaling crosstalk with cardiomyocytes, they play key roles in the development, maintenance, and remodeling of the ventricular myocardium (Miragoli et al., 2006; Souders et al., 2009; Bowers et al., 2010; Ongstad and Kohl, 2016). The use of human heart cells improves the relevance of the model system, due to identical human genetic background with a complete organotypic, however immature, protein expression pattern (Eschenhagen and Carrier, 2019). Furthermore, human cell-derived models offer the possibility of drug testing in a framework of personalized medicine (van Meer et al., 2016). 3D cell culture is motivated by the necessity to study cells in a tissue-like environment to which ECM proteins contribute a large part (Simpson et al., 1994).

CF that are remaining mostly quiescent in adult healthy tissue (Chen and Frangogiannis, 2013) become activated after enzymatic isolation from the organ and start proliferating on rigid surfaces (Serini and Gabbiani, 1999). These activated CF acquire a motile phenotype similar to smooth muscle cells and have been coined “myofibroblasts” (Tomasek et al., 2002). In practice, CF and other non-myocytes in 2D cultures become overgrown and often obstruct other cell types making it difficult to study normal function and drug effects in 2D co-culture. Alternatively, a trans-well culture with a physical separation of two cell types allows for the study of paracrine interactions but does not include direct cell-cell signaling or the presence of native ECM. CF culture in scaffold-free, multicellular aggregates does not lead to significant proliferation of primary CF, even on the surface of the spheroid, unless stimulated by growth- or other profibrotic factors [(Desroches et al., 2012; Lee et al., 2019), and own observations]. Therefore, 3D co-culture offers an option to study myocardial cells with different proliferative potential and their interactions *in vitro*, at baseline and in stress conditions.

The ratio of hiPSC-CM:CF of 4:1 was chosen for this study to represent proportions as found in healthy tissue (Banerjee et al., 2007), not to mimic a fibrotic state. For the cardiomyocyte component of the 3D co-culture we chose commercially available, pre-matured human iPSC-derived cardiomyocytes (Lapp et al., 2017). These cells form cardiac spheroids that show spontaneous contractions and respond to external electrical, pharmacological, and physical stimuli (Beauchamp et al., 2015). 3D co-culture of hiPSC-CMs and CF may improve organotypic features of this particular 3D model system, as previous studies have suggested positive effects of CF on cardiomyocytes in non-fibrotic conditions, by producing ECM and other secreted factors that affect protein expression and differentiation in the myocardium and in cultured cardiomyocytes (Eppenberger-Eberhardt et al., 1997; LaFramboise et al., 2007; Pfannkuche et al., 2010).

In the present study, we sought to advance the knowledge of hiPSC-CMs and CF in 2D-, 3D-, single-, and co-culture conditions. We have investigated spontaneous and electrically paced contractions, cytoarchitecture, and expression of selected genes related to cellular maturation and activation, electrophysiological properties, and ultrastructure after up to 1 month in culture.

## Materials and Methods

### Culture of hiPSC-CMs and CF

**Figure 1:**
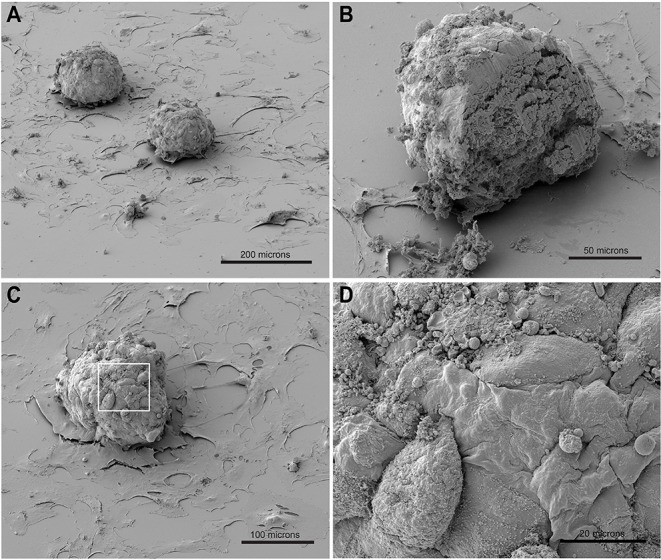
SEM of spheroids made of hiPSC-CMs and CF placed on gelatin-coated glass coverslips thereby allowing outgrowth of fibroblasts. **(A)** Two spheroids and out-growing CF on the glass surface. **(B)** Spheroid cut by scalpel after critical point drying and re-sputtering with gold. **(C,D)** Details of the spheroid surface (enlarged in **D**).

**Figure 2:**
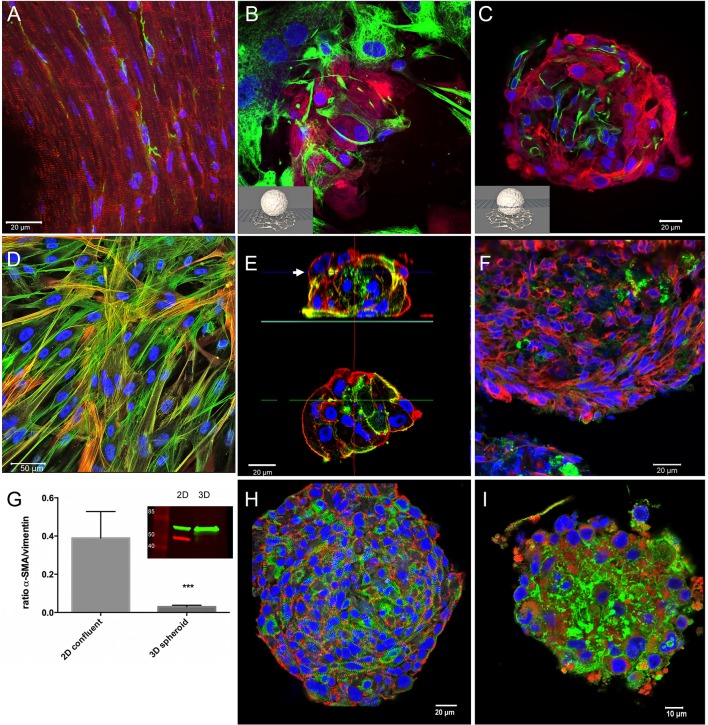
Immunohistochemical characterization of tissue, 2D-, and 3D-cultures. Nuclei are labeled with DAPI (blue) in all images. **(A)** Cryosection of an adult mouse heart immunostained for myomesin (red) and vimentin (green). **(B)** Confocal optical section on the substrate level of a spheroid made of hiPSC-CM and CF cultured for 1 month. Immunostaining for myosin heavy chain (red), and vimentin (green). **(C)** Confocal optical section above the substrate level of the same spheroid as shown in **(B)**. **(D)** 2D-cultured cardiac fibroblasts immunostained for alpha-SMA (red) and all actin (green) shown as a maximum intensity projection of optical sections. **(E)** Single confocal optical sections perpendicular to the substrate through a CF-only, small spheroid (above) cultured for 5 days and immunostained for all actin (red) and alpha-SMA (green), and an optical section at midlevel (below). **(F)** Cryosection of fibroblast-only spheroid cultured for 3 weeks immunostained for vimentin (red) and alpha-SMA (green). **(G)** Western blot experiments and quantitative assessment of the ratio of alpha-SMA/vimentin (*n* = 4, ***p < 0.001) demonstrating the difference of alpha-SMA content in 2D vs. 3D cultures of pure CF. **(H)** Whole-mount immunostaining of a cardiomyocyte-only spheroid 1 month in culture, N-cadherin (red) and EH-myomesin (green). **(I)** Cardiomyocyte-only spheroid 1 month in culture, whole-mount immunostained for all-actin (red) and laminin (green).

**Figure 3:**
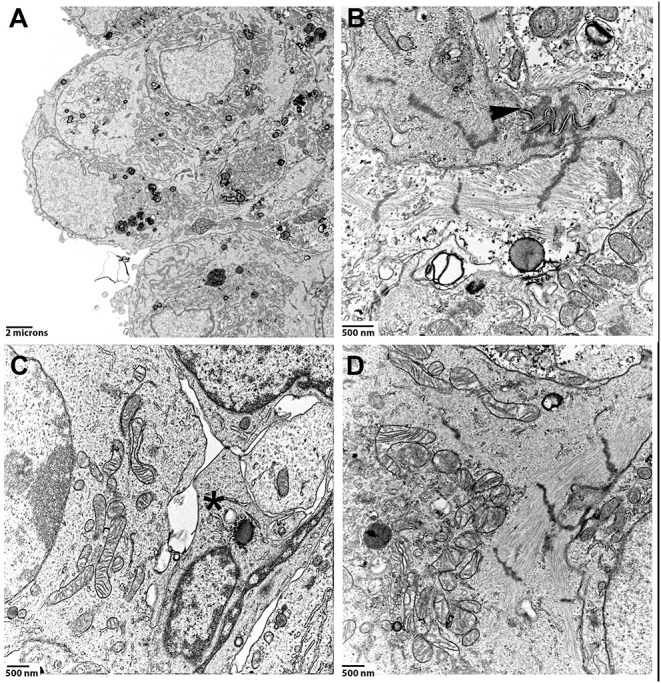
Spheroids made of hiPSC-CMs and CF were cultured for 1 month, then processed for TEM. **(A)** Low magnification image of a region close to the surface of a spheroid. **(B)** High magnification of a cell-cell contact region. Arrowhead points to a well-formed intercalated disc-like structure. Z-disc densities are found around the intercalated disc-like structure. **(C)** Inner layer of a spheroid that to shows the contact-zone between a cardiomyocyte (on the left) and non-myocytes (middle, marked by an asterisk). **(D)** Perinuclear mitochondria and myofibrils in a cardiomyocyte.

**Figure 4:**
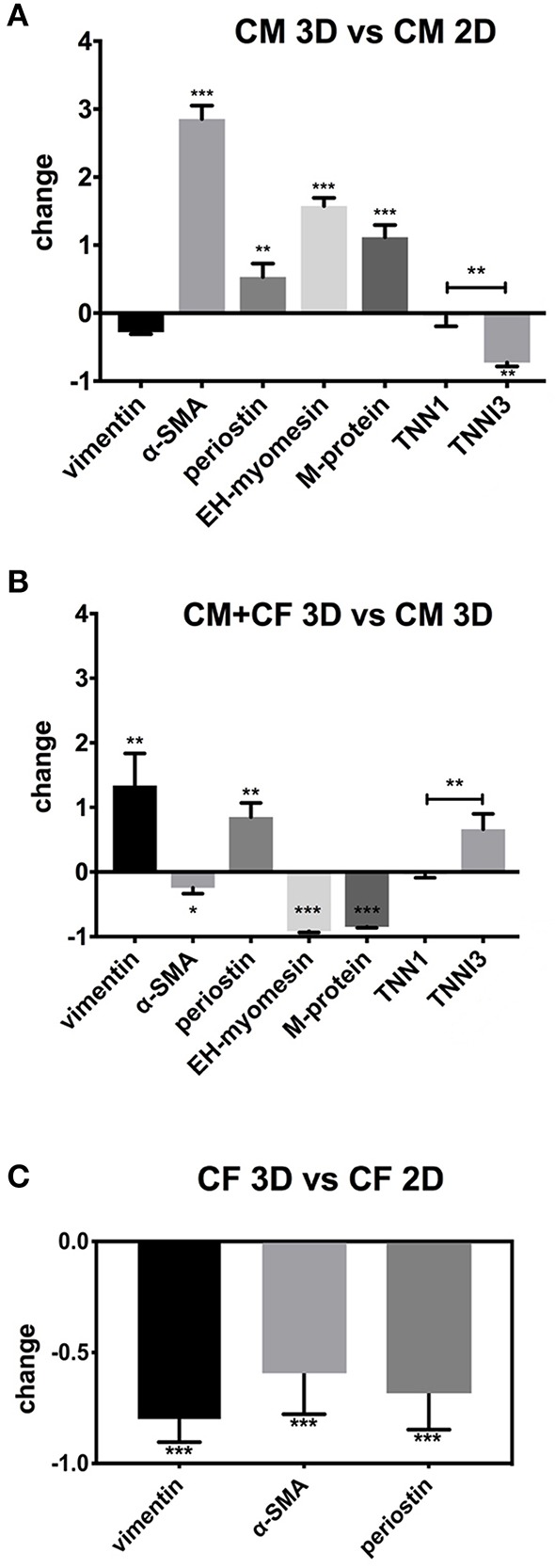
qPCR of 2D- and 3D-cultured hiPSC-CMs and CF. Shown are fold-changes of expression normalized to GAPDH expression. **(A)** Comparison of gene expression of pure hiPSC-CMs (CM) in 3D vs. 2D culture. **(B)** Comparison of co-cultured hiPSC-CMs and CF in 3D vs. pure hiPSC-CMs in 3D. **(C)** Comparison of pure CF in 3D vs. CF monolayer culture in 2D. *N* = 3, *p < 0.05, **p < 0.01, ***p < 0.001.

**Figure 5:**
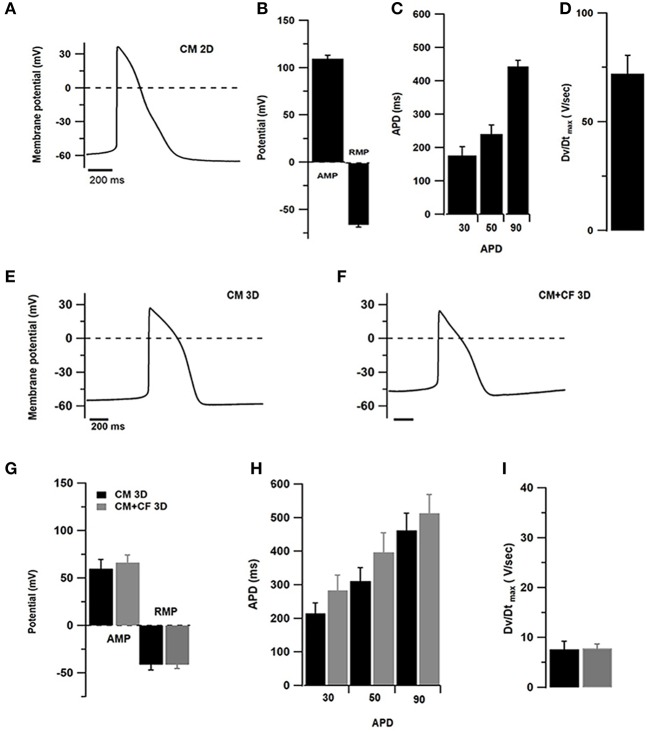
Electrophysiological measurements in 2D- and 3D-cultured hiPSC-CMs (CM) with and without CF showing spontaneous APs. **(A–D)** Patch-clamp assessments of hiPSC-CMs cultured in 2D culture for 1 week (*n* = 10 cells). **(A)** Representative AP tracings of 2D-cultured cardiomyocytes. **(B)** Amplitude (AMP) and resting membrane potential (RMP). **(C)** Action potential duration at 20, 50, and 90% of the amplitude. **(D)** Maximum upstroke velocity. **(E)** Representative AP tracing measured in pure hiPSC-CM spheroids. **(F)** Representative AP tracing from co-cultured spheroids of hiPSC-CM and CF. **(G)** Amplitude and resting membrane potential in spheroids without (black) and with CF (gray). **(H)** Action potential duration at 20, 50, and 90% of the amplitude in spheroids without and with CF. **(I)** Maximum upstroke velocity in spheroids without and with CF. *N* = 6–10 spheroids per group.

**Figure 6:**
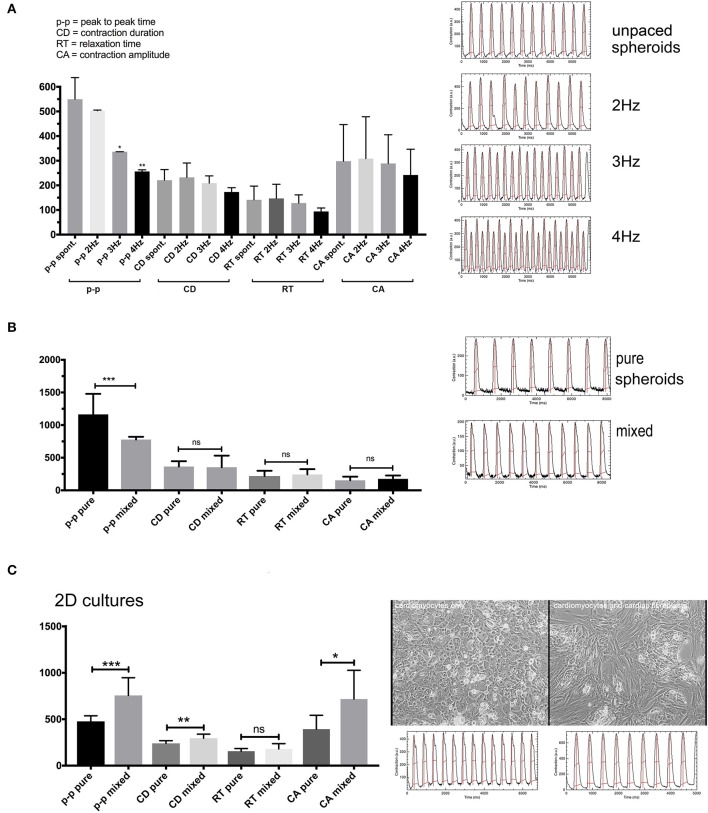
**(A)** Contraction features in co-culture spheroids 10 days in culture at different pacing frequencies. The spheroids followed pacing frequencies of 2–4 Hz reflected by the time of the “p-p” parameter since 2 Hz = 500 ms, 3 Hz = 333 ms, and 4 Hz = 250 ms. **(B)** Spontaneous contractions with spheroid of pure hiPSC-CMs or co-culture spheroids at 1 month in culture. **(C)** Spontaneous contractions in 2D cultures of hiPSC-CMS or cultures with added CF. *N* = 4–6 spheroids per group or fields in 2D-cultures, *p < 0.05, **p < 0.01, ***p < 0.001.

**Figure 7:**
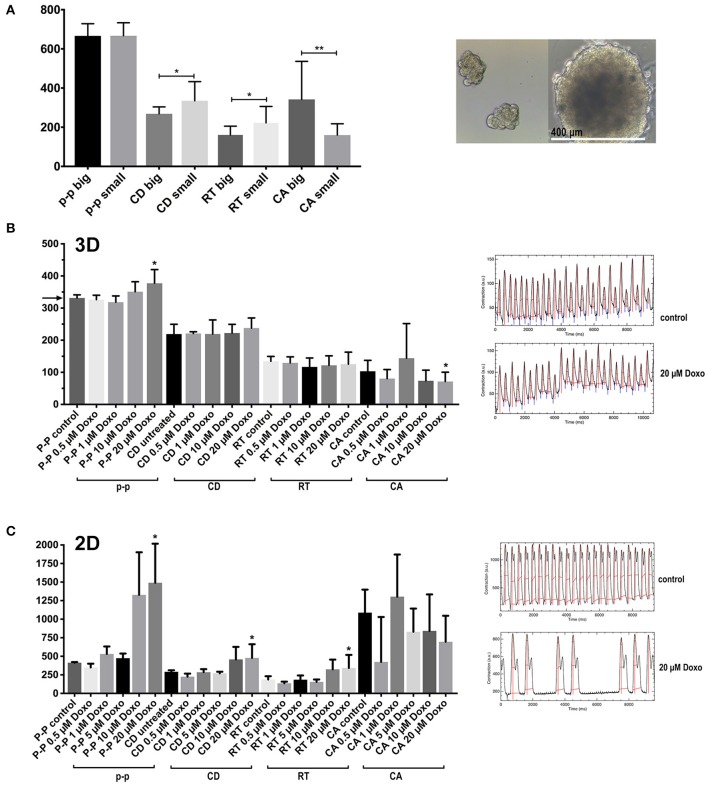
Contraction features in small and large multicellular aggregates, and effects of cardiotoxic substances. **(A)** Features of spontaneous contractions of small and large aggregates (50 ± 8 and 400 ± 47 μm in diameter) of hiPSC-CMs and CF were investigated. **(B)** The cardiotoxic cancer therapy doxorubicin (Doxo) was added to co-culture spheroids. The spheroids were electrically paced at 3 Hz (the corresponding value is indicated by an arrow in the y-axis for the p-p parameter) **(C)** The effect of Doxo on 2D cultured hiPSC-CMs. *N* = 4–6 spheroids per group or fields in 2D-cultures, *p < 0.05, **p < 0.01.

Commercially available human iPSC-derived cardiomyocytes (iCell^2^ cardiomyocytes and Cor.4U cardiomyocytes) (Figures 1–3) were obtained from Cellular Dynamics International Inc. (CDI, Madison, WI, USA) and from Ncardia (Cologne, Germany) (Figures 4–7). Differentiated iCell^2^ hiPSC-CMs are allowed to mature until day 32 at which point, they are frozen for shipping. The details of these procedures have been published previously (Babiarz et al., 2012). According to the provider, Cor.4U cardiomyocytes were purified by using puromycin selection driven by the tissue specific promoter cardiac alpha-myosin heavy chain before shipping. Cor.4U hiPSC-CMs were shipped as live culture in medium-filled flasks and passaged on arrival using Accumax (Sigma) detachment solution. Cryopreserved iCell^2^ cardiomyocytes were rapidly thawed, then diluted in iCell^2^ plating medium and seeded into Nunc culture dishes (Thermo Fisher Scientific) or microwell dishes with 7 mm diameter wells and glass bottom (MatTek corp., Ashland, USA) coated with human fibronectin (Sigma). For electron microscopy, gelatine-coating of cleaned glass coverslips was used. After 48 h, medium was changed to maintenance medium and then changed every 2 days. The cells expressed a number of cardiac markers in 2D-culture and in spheroid culture as shown previously (Beauchamp et al., 2015). Non-myocytes were virtually absent in cardiomyocyte-only cultures. Primary, human embryonic cardiac fibroblasts (CF) were obtained from ScienCell (Chemie Brunschwig, Basel, CH). CF were expanded on human fibronectin-coated cell culture flasks (Greiner BioOne) with “Dulbecco's Modified Eagle Medium” (DMEM) with low glucose and supplemented with 10% fetal calf serum and antibiotics. All cell cultures were maintained in a HeraCell (Thermo Fisher Scientific) incubator at 37°C and 5% CO_2_.

### Production and Maintenance of Cardiac 3D Cultures

For preparing co-culture cardiac spheroids, CF and hiPSC-CM were detached from 2D culture bottles, or directly thawed from cryopreserved storage vessels in case of iCell^2^ cardiomyocytes and diluted with plating medium so that a ratio hiPSC-CM:CF of 4:1 resulted. Fibroblasts selected for this study were from fetal origin and should not induce a pro-fibrotic effect in 3D co-culture (Li et al., 2017). For each spheroid 40 microliter of medium containing 5,000 cells were used for self-assembly using the GravityPlus^TM^ hanging-drop system (InSphero, Switzerland). After 4 days in the hanging drop without medium change, the spheroids were transferred to a 96-well spheroid receiver plate with non-adhesive surface (GravityTRAP^TM^, InSphero, Switzerland) in a volume of 70 μL maintenance medium per well-supplied by the manufacturer of the hiPSC-CMs. The spheroids were then cultured up to 30 days, or as indicated in the respective result section, with medium changes every 2 days. The alpha1 adrenergic agonist phenylephrine (PE) and 30 μM ascorbic acid (Sigma) was added to the culture medium at a final concentration of 100 μM in order to enhance myofibrils in cardiomyocytes (Ehler et al., 1999; Foldes et al., 2011). For a series of experiments with spheroids of different sizes, ultra-low attachment plates with microwells were used for the production of smaller cellular aggregates according to protocols supplied by the manufacturer (Sphericalplate 5D, Kugelmeiers AG, Zollikerberg, Switzerland).

### Video-Analysis of Monolayer and Spheroid Beating Activity

A modified GoPro HeroBlack 6 camera (Back-Bone Gear Inc., Kanata, Canada) was used to record short video sequences at high frame rate, 240 frames per second, of spheroids in a heating chamber with warmed lid and temperature controller (Ibidi, GmbH, Martinsried, Germany) on the stage of an inverted microscope (Nikon Eclipse TE2000-U) with a Nikon Plan Fluo 10×/0.3 phase contrast lens. Cultures were allowed to warm up to 37°C for 10 min and DMEM containing 25 mM HEPES buffer was used for recordings. For some experiments, a self-made pacing chamber with platinum wires inserted in a glass-bottom dish was used with a MyoPacer (Ionoptix, USA), set to different field-pacing frequencies at 10 V, bipolar pulses of 4 ms duration. The open-source macro “Musclemotion” for ImageJ by Sala et al. (2017) was then used on an Apple MacPro equipped with 64 GB RAM to extract motion data from videos after format conversion and data reduction steps. This software has been validated with a number of cardiac models where beating activity is observed and we have additionally verified the software in our lab by using freshly isolated adult rat ventricular cardiomyocytes that precisely follow an electrically induced contraction frequency (Supplementary Movie 1). For 3D cultures, videos were used for the analysis that show the entire circumference of the spheroids. For 2D cultures, regions of interest were defined that comprised single cells or small groups of cells.

### Immunocytochemistry and Microscopy of 2D-Cultured Cells, Whole-Mount Spheroid Preparations, and Cryosections

2D-cultured cells fibronectin-coated polystyrene culture dishes (Nunc) were washed with PBS then fixed with 3% para-formaldehyde in PBS for 15 min, permeabilized with 0.2% Triton-X100 (Sigma) in PBS for 10 min, incubated for 30 min with bovine serum albumin (Sigma) 1 mg/mL in PBS at room temperature, incubated over night with primary antibodies at 4°C, washed three times with PBS and incubated 1 h with secondary antibodies goat anti mouse or anti rabbit coupled to Alexa fluorescent dyes (Invitrogen). DAPI (Sigma) was added to secondary antibodies to visualize the nuclei or Vectashield mounting medium with DAPI was used (Vectorlabs). Preparations were examined on a Zeiss LSM 710 confocal microscope using 40× and 63× Zeiss oil immersion lenses (Carl Zeiss). For whole-mount immunostaining of entire spheroids, live spheroids either were allowed to adhere overnight onto fibronectin-coated glass-bottom dishes (MatTek) or 10–20 spheroids were collected by sedimentation and further incubation and washing steps were executed in 500 μL Eppendorf-tubes. After fixation with 3% para-formaldehyde (PFA) for 1 h in the cold, permeabilization and antibody incubation steps in 1% BSA/PBS/10% Tween-20 (Sigma) were prolonged to 1 day each in the cold before examination by confocal microscopy using a 20× air lens. For cryosections, spheroids were collected by gentle spinning in a tube. The spheroids were then fixed with 3% para-formaldehyde in PBS for 60 min, washed with PBS, stored and embedded in OCT compound. Blocks were cut using a Zeiss Hyrax cryostat. Frozen sections on slides were air dried for at least 1 h and then post-fixed in cold acetone. After a blocking step with 1% BSA/PBS, primary antibodies in 1% BSA/PBS, and 0.3% Tween20 were applied overnight. Further processing was the same as with 2D cultures. A sample of left ventricular, normal adult mouse heart was a gift from Maria Essers (Institute for Biochemistry and Molecular Medicine, Bern University) that was fixed with PFA, and used for cryosections and immunostaining as detailed above for 2D cultured cells.

### Western Blotting

Cells and spheroids were lysed in cold lysis buffer containing 3.7 M urea, 134.6 mM Tris, 5.4% SDS, 2.3% NP-40, 4.45% beta-mercaptoethanol, 4% glycerol, 6 mg/100 ml bromophenol blue (all from Sigma) according to Ehler et al. (1999). Cell lysates were further treated by repeated expelling through a fine needle and boiled for 5 min, then separated by SDS-PAGE using precast 4–20% gradient gels (BioRad) and blotted to Protran BA 83 nitrocellulose membranes (Whatman). Immunodetection was carried out after blocking in 5% milk in TBST (20 mM Tris base, pH 7.5, 150 mM NaCl, 0.05% Tween-20). For the visualization of signals, a LiCor Odyssey infrared imaging system (LiCor) was used with secondary antibodies coupled to Alexa700 and Alexa790 fluorescent dyes (ThermoFisher).

### Antibodies

Antibodies used to detect proteins by Western blotting and immunofluorescence staining were monoclonal mouse antibodies (mAb) to alpha-SMA clone 1A4 (Sigma A2547), monoclonal rabbit antibodies to vimentin (Novus biologicals NBP1-40730), mAb to beta myosin heavy chain (LifeSpan BioSciences, clone IML-64, LS-C312448), polyclonal rabbit antibodies (pAb) to all-actin (Sigma A2066), pAb to laminin (Sigma L9393), mAb to N-cadherin clone 13A9 (Novus biologicals NBP1-48309), pAb to connexin-43 (Abcam, ab11370), and pAb produced in rabbits to embryonic heart-specific human myomesin isoform huEH-myomesin (Agarkova et al., 2000).

### Electrophysiological Measurements

HiPSC-CMs were passaged at low density and seeded on fibronectin-coated glass-bottom dishes (Mattek) for 2D culture or used for the production of spheroids with and without CF in hanging drops, cultured for 10 days in GravityTrap and then allowed to adhere to glass bottom dishes for another 5 days. Spontaneous action potential recordings were performed on either 2D-cultured hiPSC-CM or spheroids in current clamp mode using using MultiClamp 700B (Axon Instruments, CA, USA) controlled by Clampex 10 (Axon Instruments, CA, USA) via a Digidata 1332A (Axon Instruments, CA, USA). Spontaneous APs in 2D-cultured hiPSC-CMs were recorded using the perforated patch clamp configuration. The patch pipettes were backfilled with amphotericin B (225 μg/mL) (Merck) and intracellular solution containing (mmol/L) 120 KCl, 1.5 CaCl_2_, 5.5 MgCl_2_, 5 Na_2_ATP, 5 K_2_-EGTA, and 10 HEPES, pH 7.4 (KOH). Spontaneous APs from intact spheroids were recorded by impaling with sharp borosilicate glass microelectrodes having pipette resistance of 20–30 MOhm (when filled with 3M KCl). 2D-cultured hiPSC-CM and spheroids were bathed in (mmol/L) 140 NaCl, 5.4 KCl, 1.8 CaCl_2_, 1.2 MgCl_2_, 10 HEPES, and 5 glucose, adjusted to pH 7.4 (NaOH). These experiments were performed at room temperature. For reducing motion artifacts, the electromechanical uncoupler blebbistatin (25 μM, Merck) was freshly added to the extracellular solution.

### Transmission Electron Microscopy (TEM)

Prior to electron microscopy, groups of 32 co-culture spheroids were collected by mild centrifugation, fixed in 4% glutaraldehyde in phosphate buffer and stored at 4°C. Samples were dehydrated, post-fixed with 1.0% osmium tetroxide, epoxy-embedded, and hardened so that the spheroids stayed sedimented at the bottom of a cone-shaped block, and cut using a Reichert Ultracut-3 microtome. Sections on copper grids were post-stained with 2.0% uranyl acetate and Reynolds lead citrate. Sections were examined using a FEI Tecnai T12 TEM (120 kV LaB6 source) electron microscope and AMT XR41-S side mounted 2K X 2K CCD camera. Image contrast enhancement and sharpening operations were performed equally to all images using Photoshop (Adobe Systems, San Jose, USA).

### Scanning Electron Microscopy

Co-culture spheroids cultured in GravityTrap for 10 days were allowed to adhere to cleaned and gelatine-coated glass coverslips for 2 days. Glass cover slips were washed in 1×PBS and fixed in 4% glutaraldehyde in phosphate buffer and stored at 4°C. Samples were dehydrated in increasing concentrations of ethanol and transferred for critical point drying in a Tousimis Autosamdri 815 (Maryland, US). Dried samples were coated with 10 nm of Au in a Leica EM ACE600 double sputter coater. Prepared samples were imaged using secondary electrons in a FEI Nova Nano SEM 230 (field emission gun) equipped with digital camera. Image contrast was adapted using Photoshop (Adobe Systems, San Jose, USA).

### RNA Isolation and Quantitative PCR

RNA isolation and cDNA synthesis: hiPSC-CMs or CF cells seeded in 2D flask culture or 3D spheroids were harvested by mechanical scraping and centrifugation. For 2D-cultured hiPSC-CM”s one million cells were used in each experiment, ~2 million CF in 2D cultures, and 48 spheroids of each 5,000 cells (total of 240,000 cells) for the 3D cultured hiPSC-CMs, CF, and mixed spheroids. Lysates were immediately processed for RNA isolation with the Quick-RNA MicroPrep Kit (Zymoresearch, Lucerna-Chem, Lucerne, Switzerland) according to the manufacturer's instructions without using DNAse. The concentration and purity of RNA was determined by the optical density (OD) value at 260 and 280 nm using an ultraviolet spectrophotometer. The purified RNA was stored at −80°C. Reverse transcription into complementary DNA (cDNA) was performed using Impron-II Reverse Transcription System (Promega) following the manufacturer's instructions using 1 μg of total RNA per reaction for all groups. qPCR was conducted using an ABI 7500 Real-Time PCR system (Applied Biosystems, Foster City, CA, USA). The reaction system was 10 μL in volume, which consisted of 5 μL of 2× real time PCR buffer Absolute SYBR Green Mix, low ROX (Thermo Scientific), containing 3 mmol/L, and ROX as a passive reference dye for normalization of data), 0.5 μL of each forward and reverse specific primers (5 mmol/μL), 3.5 μL of RNase-free ultrapure water, and 0.5 μL of cDNA template. The reaction conditions were as follows: pre-denaturation at 95°C for 10 min, followed by 40 cycles of denaturation at 95°C for 15 s, annealing at 60°C for 30 s and elongation at 72°C for 30 s. All reactions were run in triplicate. A table of CT values for each PCR run is available in Supplementary Materials. Relative changes in respective mRNA expression were determined by the 2-ΔΔCt method (Pfaffl, 2001), normalized to the reference GAPDH gene expression.

### Statistical Analysis

All values are expressed as mean ± S.D. Statistical analysis of differences observed between the groups was performed by Student's unpaired *t*-tests. Statistical significance was accepted at the level of *p* < 0.05. GraphPad Prism 7.0 (GraphPad Software, San Diego, USA) was used for statistics and graph production.

## Results

### General Morphology and Inner Structure of Co-culture Spheroids

The morphology of entire spheroids was examined by SEM (Figure 1). Co-culture spheroids made of hiPSC-CMs and CF were produced in hanging drops, cultured for 10 days in GravityTrap multi-well plates and then allowed to adhere to gelatine-coated glass coverslips in medium-filled 35 mm dishes for 2 days. In this time the spheroids became firmly attached to the glass coverslip while some CF started growing out. The cardiac spheroids did not flatten out during the time cultured on the adhesive surface, in contrast to spheroids made of only CF (not shown). Outgrown cells were thinly spread out on the flat substrate, while those cells seen on the surface of the spheroid kept a rounded or spindle shape (Figure 1D). It is possible to make use of outgrowing cells for testing pharmacological compounds, as others have demonstrated (Christoffersson et al., 2018), however, we have put emphasis on spheroids and defined 2D cultures in this study. Opening a spheroid by manually cutting it with a blade provided an opportunity to inspect the interior structure (Figure 1B). The cut face revealed that the spheroid was entirely filled with compact cellular material and no signs of blebbing or vacuole formation could be seen. In phase contrast light microscopy, spheroids made of hiPSC-CMs and CF appeared to have a smoother surface and to be more spherical than those made of hiPSC-CMs only (see Supplementary Movie 2), which might be caused by layers of protein deposition on the surface. However, SEM inspection suggested that the surface of these co-culture spheroids is not covered with material and the cells are directly exposed to the liquid medium of the culture, which later was confirmed by ultrathin sections and TEM (Figure 3A).

### Immunocytochemical Characterization of Cardiomyocytes and CF *in vivo*, 2D Culture and in Single and Co-cultured Spheroids

For the identification of hiPSC-CMs in 2D- and 3D cultures we used sarcomeric proteins that are well-organized at this stage in hiPSC-CM in 2D culture and in cardiac spheroids (Beauchamp et al., 2015; Zuppinger et al., 2017). The intermediate filament protein vimentin was used as marker for cardiac fibroblasts because it is expressed in both quiescent and activated cardiac fibroblasts *in vitro* and *in vivo* (Ali et al., 2014; Kofron et al., 2017). Vimentin is not found in freshly isolated adult rat cardiomyocytes, but it is expressed to some extent in 2D cultured hiPSC-CMs as we have previously shown (Zuppinger et al., 2017). Using cryosections of adult murine left ventricle we examined the distribution and morphology of cardiac fibroblasts in normal murine ventricular tissue (Figure 2A). The cells presented a comparably small cell body and nucleus with thin longitudinal extensions between the cardiomyocytes *in vivo*. We then investigated the distribution and morphology of CF in flat and 3D culture.

For this aim, cardiac spheroids cultured for 1 month were placed on glass bottom dishes allowing the outgrowth of CF on the glass surface (Figures 2B,C). We fixed the culture and immunostained it for vimentin and beta-myosin heavy chain and recorded optical sections using confocal microscopy on the substrate level (Figure 2B) and above (Figure 2C). The fibroblasts growing at low density on the flat surface exhibited the typical phenotype of a polygonal, spread-out cell body filled with vimentin filaments (Figure 2B), eventually assuming an elongated shape marked by actin-stress fibers in confluent cultures (Figure 2D). However, in 3D conditions, the CF appear to assume a similar morphology as in ventricular tissue with a small cell body and nucleus (Figure 2C). No overlap of vimentin and beta-myosin heavy chain signals was observed. The fibroblasts were interspersed among cardiomyocytes, although larger accretions of CF were also observed. CF did not form a closed layer on the surface of co-culture spheroids (not shown).

We then assessed 2D cultures and spheroids made only of CF and used the myofibroblast marker alpha-smooth muscle actin (alpha-SMA) (Clément et al., 2005) in different culture types (Figures 2D–G). In 2D culture CF quickly formed a dense monolayer of partially overgrowing cells. Immunostaining for all-actin and alpha-SMA showed prominent actin stress-fibers in all cells with varying degrees of alpha-SMA labeling in individual cells (Figure 2D). We then produced small multicellular aggregates and larger spheroids made of CF previously cultured in monolayers and used whole-mount immunolabeling (Figure 2E) or cryosections (Figure 2F) to examine cellular morphology and the myofibroblast marker alpha-SMA. In small aggregates of CF cultured for 5 days, actin stress-fibers were absent, and instead actin was found in form of an actin cortex lining the cell membranes (Figure 2E). The immunolabeling for all-actin and alpha-SMA showed a heterogeneous distribution of alpha-SMA with the myofibroblast marker only in a minority of cells. However, in larger CF-only spheroids cultured for 3 weeks, alpha-SMA was found only in traces (Figure 2F). In order to quantify alpha-SMA expression we did western blotting of lysed monolayer-cultured CF and pooled CF spheroids cultured for 3 weeks (Figure 2G). The results consistently demonstrated a strong downregulation of alpha-SMA, but not vimentin, in 3D culture. In spheroids made only of hiPSC-CMs we observed by whole-mount immunolabeling after 1 month in culture that the cardiomyocytes assumed a spindle-shaped morphology, with abundant N-cadherin positive cell-cell contacts and myofibrils marked by EH-myomesin in the absence of stress fibers (Figure 2H). Connexin-43 indicating gap junctions was detected by whole-mount immunolabeling in co-culture spheroids (not shown) though at low abundance in both the cardiomyocyte and CF portions of the spheroids, similarly to previous findings in cardiomyocyte-only microtissues (Beauchamp et al., 2015). Typically, cells were found aligned in a linear fashion following the curvature of the spheroid in the outer layers (lower right in Figure 2H), while cells toward the center of the spheroid showed a more random orientation. Immunolabeling for all actin and for laminin demonstrated ample expression of this extracellular matrix protein in the spheroid around and in-between the hiPSC-CMs (Figure 2I).

### Ultrastructure of Spheroids Made of hiPSC-CM and CF Cultured for 1 Month

Co-culture spheroids made of hiPSC-CMs and CF for a total of 5,000 cells were produced in hanging drops, cultured for 30 days in GravityTrap with frequent medium changes, and then fixed and processed for TEM. Inspection of areas including the spheroid surface revealed that the cells are directly exposed to the culture medium and single cells appear tightly packed in this region of the spheroid (Figure 3A). We found cytoskeletal elements and organelles typical for cardiomyocytes such as myofibrils, many mitochondria and cell-cell contact structures, i.e., adherens junctions and desmosomes reminiscent of the interdigitating structures of the intercalated disc in the myocardium (Figures 3B,D). The orientation of these elements varied, and myofibrils were often only seen truncated in the ultrathin sections. Those sarcomeres that were visible in their entire length did not show differentiated M-bands (Figure 3D). The identification of non-muscle cells on TEM-images of these spheroids is more difficult than in the mature myocardium, where the cardiomyocytes assume a strictly cylindrical morphology and generally are filled with myofibrils. Nevertheless, based on the assumption that hiPSC-CM and CF differ in ultrastructural criteria such as presence of myofibrils, density of perinuclear mitochondria and cell size, we propose that a situation shown in Figure 3C demonstrates cell-cell contacts between non-myocytes and hiPSC-CMs. These contacts appear to form punctate cell-cell interactions (membrane extensions) similar to those that have been recently described between cardiomyocytes and non-myocytes in tissue using TEM and electron-tomography (Quinn et al., 2016). Mitochondrial cristae (Figures 3C,D) were similar to those found in immature cardiomyocytes typically showing a lower density than those in the adult myocardium (Hilse et al., 2018).

### Gene Expression of 2D-/3D-Cultured hiPSC-CMs, 3D-Co-Cultures and 2D-/3D Cultured CF

Gene expression was examined under five conditions: pure hiPSC-CMs in 2D culture for 3 weeks, pure hiPSC-CMs in 3D culture for 3 weeks, hiPSC-CMs and CF in 3D co-culture for 3 weeks, pure CF in 2D culture for 1 week, and pure CF in 3D culture for 3 weeks. We kept the culture period of CF in 2D culture shorter than for the spheroids because these cells tended to die and lift off the plate after longer culture times at confluence. Gene expression analysis was performed to investigate myocardial maturation markers (EH-myomesin/*MYOM1*, M-protein/*MYOM2*, ssTnI/*TNNI1*, cTnI/*TNNI3*, alpha-SMA/*ACTA2*) and markers of the activation status of CF, i.e., the myofibroblast phenotype (alpha-SMA/*ACTA2*, periostin/*POSTN*, and vimentin/*VIM*) in 2D- and 3D-cultured cells. Results were normalized to GAPDH expression. hiPSC-CMs showed a lower expression level of *TNNI3* (−70%) when cultured in 3D in comparison with the same cells cultured in a monolayer for the same time, but an increase in expression of *MYOM1* and *ACTA2* (2.5–4-fold; Figure 4A) in 3D was found. In contrast to this, the analysis of gene expression in mixed spheroids hiPSC-CMs with CF showed a lower expression of *ACTA2* compared to 3D culture of pure hiPSC-CMs (Figure 4B). In addition, *MYOM1* and *MYOM2* (−90%) were down-regulated, but *TNNI3* was comparably more expressed than *TNNI1* in the presence of CF. Pure CF cultured in 3D condition showed decreased expression of all three investigated genes compared to 2D condition, from 50% less expression in the case of *POSTN* to 90% for *ACTA2* and *VIM* (Figure 4C). See Table 1 for primers sequences.

**Table 1:** Primer sequences used for qPCR of 2D- and 3D-cultured human cardiac cells.

Glyceraldehyde-3-phosphate-Dehydrogenase (*GAPDH*)	Forward	ATGGAAATCCCATCACCATCTT
	Reverse	CGCCCCACTTGATTTTGG
Vimentin (*VIM*)	Forward	CGTCCACACGCACCTACAG
	Reverse	GGGGGATGAGGAATAGAGGCT
alpha-smooth muscle actin (alpha-SMA)(*ACTA2*)	Forward	GGGTGATGGTGGGAATGG
	Reverse	GCAGGGTGGGATGCTCTT
Periostin (*POSTN*)	Forward	TGCCCAGCAGTTTTGCCCAT
	Reverse	CGTTGCTCTCCAAACCTCTA
EH-Myomesin (*MYOM1*)	Forward	GAGCGATGAGCCTGGTGGACTA
	Reverse	AGAACCATTGAGTCACGAAAAC
M-protein (*MYOM2*)	Forward	CACAGAGAGCCTCCAGCCAGAC
	Reverse	CCGCTCTTCAAATGTGTGTCTC
slow skeletal troponin-I (ssTnI) (*TNNI1*)	Forward	GTGGGTGACTGGAGGAAGAA
	Reverse	GTGAGCTGGGTTGGAGAAGA
Cardiac troponin-I (cTnI) (*TNNI3*)	Forward	CACCTCAAGCAGGTGAAGAAG
	Reverse	CAGGAAGGCTCAGCTCTCAA

### Electrophysiological Characterization of 2D-/3D-Cultured hiPSC-CMs and Mixed Spheroids

After 1 week, 2D cultures of pure hiPSC-CMs contained several islands of cells showing spontaneous beating activity. For 2D-cultured hiPSC-CMs perforated patch current-clamp has been used and impaling with sharp microelectrodes of outer layers of cardiac spheroids in 3D cultures (Figure 5). To compare spontaneous action potentials (AP), only recordings of cells with ventricular-type AP were considered, although other patterns indicating the presence of nodal or atrial sub-types were also observed (not shown). The resting membrane potential (RMP) of hiPSC-CMs cultured in 2D culture was found to be at −65.9 ± 8 mV and in 3D cultures of pure hiPSC-CMs at −41.4 ± 13 mV and −41.3 ± 11 mV in 3D co-cultures with CF (Figures 5B,G). AP amplitude was found to be at 109.7 ± 10 mV in 2D-cultured hiPSC-CMs, 59.8 ± 23 mV in 3D cultured pure hiPSC-CMs, and 66.3 ± 21 mV in 3D co-cultures (Figures 5B,G). Action potential duration (APD) was measured at 30, 50, and 90% of the AP peak in 2D and 3D cultures (Figures 5C,H). In addition, we also compared the AP parameters with or without addition of CF to spheroids (Figures 5E,F). Assessment of different AP parameters resulted in a depolarized RMP around −40 mV and slow upstroke velocity at 10 V/s (Figures 5G,I) compared to 2D culture. The APDs were measured at 30, 50, and 90% of the AP peak in 3D culture with or without CF (Figure 5H).

### Assessment of Contractile Motion in 2D- and 3D-Culture by Computational Video Analysis

Spheroids made of hiPSC-CMs and CF, or hiPSC-CMs alone, were produced in hanging drops as described. Additionally, hiPSC-CMs were cultured in 2D culture with and without adding CF in a later step, and ultra-low attachment plates were used to produce smaller spheroids. All measurements were performed at 37°C and after a warm-up time of 10 min. Adequate temperature regulation is crucial for any experiments involving spontaneous contractions in hiPSC-CMs as we have found in a previous study (Beauchamp et al., 2015). Stability of spheroid contractile function has been shown for more than 100 days (Fleischer et al., 2019). As the video analysis is based on the full image comprising the entire spheroid, it takes into account motion in all directions. The following contractile motion parameters were extracted from image sequences by computational video analysis and shown in Figures 6, 7: peak to peak time (p-p), contraction duration (CD), relaxation time (RT), and contraction amplitude (CA). Electrical field pacing is useful for revealing functional alterations that are associated with drug treatments or genetic conditions. We have previously used this method with freshly isolated adult cardiomyocytes and in long-term cultured cardiomyocyte monolayers treated with cancer therapies and growth factors (Timolati et al., 2006, 2009). Therefore, we investigated the spontaneous rate, but also the effect of increasing pacing frequencies on co-culture spheroids to evaluate the outcome of electrical pacing on contractile motion parameters in this model (Figure 6A).

Spontaneous rates were between 2 Hz in earlier cultures (Figure 6A) and around 1 Hz in 1 month old spheroids without CF (Figure 6B). Most spheroids followed electrical field pacing frequencies in the range of 2–5Hz (Supplementary Movie 2). Pacing frequencies below 2 Hz were not effective as the spontaneous activity persisted around 2 Hz and additional pacing led to irregular beating patterns (not shown). The other parameters CD, RT, and CA only showed a trend to smaller values with increasing pacing frequency (Figure 6A). Occasionally, an alternating pattern in contraction amplitudes was observed that became more pronounced with higher pacing frequencies (Figure 6A). We then measured the spontaneous contractions of spheroids cultured for 1 month and made of only hiPSC-CMs or co-cultured spheroids (Figure 6B). Only the p-p parameter was different between the two types of spheroids, showing faster baseline rate of contraction in spheroids with 25% CF added. We then investigated the outcome of adding CF to hiPSC-CM in 2D culture. For this experiment the hiPSC-CM were seeded first at low density, then CF were added to the cardiomyocyte culture and CF filled all gaps between the myocytes. After another week of 2D-culture, contractile motion in regions of interest was assessed without external pacing in pure and mixed cultures (Figure 6C). In this context, the cardiomyocytes showed slower spontaneous contractions. CD and CA parameters also differed between the two culture types (Figure 6C).

We compared spheroids of different size, on average 50 ± 8 μm and 400 ± 47 μm in diameter (Figure 7A) to investigate if the size of the spheroid and the suspected development of hypoxic zones affected overall contractile motion. Both types of spheroid were made in parallel and with the same mixed cell suspension of hiPSC-CMs plus CF. The large spheroids were made in hanging drops while the small ones were made in non-adhesive microwells. The shape of the small aggregates was not entirely spherical and appeared less densely packed. Visualization of intracellular calcium cycling showed regular signals in standard-sized spheroids but revealed non-synchronized calcium release events in small multicellular aggregates (Supplementary Figure 1 and Supplementary Movies 3, 4). Interestingly, the size difference did not affect the rate of spontaneous beating as shown by the p-p parameter. While kinetics parameters indicated faster speeds of contractions in large spheroids, the measured contraction amplitude was also higher in the larger tissues (Figure 7A). We then tested the effect of cardiotoxic substances on co-culture spheroids using the cancer therapy doxorubicin (Doxo) that shows a well-described, wide range of cardiotoxic effects on cardiomyocytes including topoisomerase inhibition, increased intracellular calcium, cytoskeleton degradation, and mitochondrial toxicity (Lim et al., 2004; Kloss et al., 2008; Chiusa et al., 2012). Doxo has been used before as a positive control for assessment of cardiotoxicity in cardiac spheroids (Beauchamp et al., 2015; Polonchuk et al., 2017; Sirenko et al., 2017). Since we had previously observed that spontaneous contractile motion sometimes stopped altogether in Doxo-treated spheroids, we challenged all the spheroids by electrical pacing at 3 Hz while recording the video after Doxo treatment (Figure 7B). The electrical pacing interval of 333 ms was reflected in the p-p parameter of untreated spheroids as expected (indicated by an arrow on the y-axis in Figure 7B). Concentrations of Doxo that had resulted in extensive myofibrillar damage and cell death in previous studies in 2D-cultured adult rat cardiomyocytes (Dimitrakis et al., 2012) had little impact on contractile motion parameters of spheroids (Figure 7B). Only at 20 μM Doxo the parameters p-p and CA showed significant changes compared to untreated spheroids. In monolayers, the effects of the treatment became evident at lower doses, but the video analysis showed higher variability than in spheroids (Figure 7C).

## Discussion

### Cytoarchitecture, Gene Expression, and Maturation

We and others have found that scaffold-free 3D culture has a significant impact on myocardial cells concerning their morphology, cytoskeletal organization, gene expression, and function. CF cultured on a rigid glass or plastic surface showed alpha-SMA-positive stress fibers i.e., prominent bundles of filamentous actin and associated proteins, which is a typical feature in mesenchymal-derived cells cultured in monolayers (Serini and Gabbiani, 1999). Taking a cell population already expressing alpha-SMA into spheroids, a marker of myo-fibroblast differentiation, led to a loss of stress fibers after a few days in CF-only spheroids while alpha-SMA protein persisted in the cortex on the inner face of the cell membrane. The entire cell morphology changed from spread-out thin cells to spherical cells and, in co-culture spheroids, to a more elongated shape between the cardiomyocytes reminiscent of the morphology found in tissue sections of the adult heart. In older CF-only spheroids, an almost complete loss of alpha-SMA was observed as demonstrated by immunostaining, western blotting, and qPCR. This change in alpha-SMA expression is likely triggered by cellular sensing of the stiffness of the environment—as it occurs in scar tissue in organs—but could also depend on locally released cytokines and the developmental age of the cells (Ali et al., 2014).

Experiments with tunable hydrogels of different stiffness (using methacrylated gelatin, collagen, and other materials) have demonstrated the importance of mechanical factors modulating CF phenotypes in 3D culture models (Hutson et al., 2011; Sadeghi et al., 2017). The non-structural ECM protein periostin is a target of transforming growth factor-β1 (TGF-beta) signaling via canonical pathways in the heart, where this protein is found in the developing myocardium playing a profibrogenic role, but not in the adult heart at baseline (Landry et al., 2017). Periostin expression followed the downregulation found for alpha-SMA in CF-only spheroids, but not in 3D co-cultures. This gene was also expressed in cardiomyocytes-only spheroids and in monolayers. Its expression may correlate with increased production of ECM proteins in 3D conditions (Norris et al., 2009). The intermediate filament protein vimentin was also found downregulated in 3D conditions, but in contrast to alpha-SMA, vimentin-positive filaments were detected by immunostaining in monolayers and in spheroids of all ages. Our interpretation of the findings shown here is that cardiac myo-fibroblasts revert to the non-activated form of CF in the spheroids, similar to the state of most CF in the healthy heart.

Gene expression changes in hiPSC-CMs showed an increased expression of alpha-SMA in 3D compared to monolayer culture. However, stress fiber-like structures were not found in 3D-cultured hiPSC-CMs. In a study using human embryonic stem cell-derived cardiomyocytes, alpha-SMA and vimentin was used as a non-cardiac marker in spheroid cultures (Fleischer et al., 2019). We have previously found both proteins in a patchy distribution in 2D-cultured hiPSC-CMs and considered this expression an immature feature of those cells (Zuppinger et al., 2017). Vimentin was not detected in cardiomyocytes in co-culture spheroids here, as the protein did not co-localize with a sarcomeric marker. Generally, the expression of alpha-SMA is found in differentiating cardiomyocytes and as a marker of myocardial hypertrophy in the adult heart (Schwartz et al., 1992). In young rats, it was found that cardiomyocytes of animals up to 2 weeks old still contain a considerable amount of alpha-SMA, but this almost completely disappears at 4 weeks (Pöling et al., 2012). Alpha-SMA re-appears in 2D cultures of adult ventricular rat cardiomyocytes cultured with fetal calf serum and treated with basic fibroblast growth factor (bFGF), similar to cellular expression patterns in the hypertrophied myocardium (Harder et al., 1998). In summary, alpha-SMA can be considered part of an immature expression pattern in cardiomyocytes, and this marker may be instrumental in assessing maturation in 3D cultures subjected to various maturation strategies.

The expression of troponins has been used to measure differentiation of cardiomyocytes, namely by measuring the ratio of the embryonic slow skeletal troponin-I (ssTnI) and the adult cardiac troponin-I (cTnI) that replaces the immature isoform (Bedada et al., 2014). In pure hiPSC-CMs 3D cultures, the mature isoform cTnI was less expressed compared to monolayer culture but increased in 3D co-culture compared to 3D monoculture indicating an improved tissue-like expression pattern in 3D co-culture with CF. For M-line proteins, EH-myomesin in the heart is downregulated after birth in vertebrates, while M-protein gets upregulated (Agarkova et al., 2000). Using immunocytochemistry, EH-myomesin protein was detected in cardiac spheroids and 2D cultures of hiPSC-CMs as previously shown, but not in fully differentiated cells such as freshly isolated adult cardiomyocytes (Zuppinger et al., 2017). We have observed here by qPCR higher expression of EH-myomesin in hiPSC-CMs 3D cultures than in monolayers, but less so when CF were present in the spheroids. Using TEM, we did not find discernable, electron-dense M-bands in the cardiomyocytes of 1-month co-cultured spheroids indicating a generally low level of cellular maturation.

Regarding the general myofibrillar content of individual cardiomyocytes in 3D cultures and the alignment of the cells, we noticed outer cell layers showing aligned cells following the curvature of spheroid to some degree, as determined by the immunostaining of sarcomeric proteins, but not in the center of the spheroid where more random orientation was observed. Commonly we found that particular regions of cardiac spheroids beat stronger than others. We speculate that those parts of the spheroids that contain aligned cardiomyocytes rich in myofibrils contribute most to the visually apparent motion. We postulate that the individual cells in the microtissues are mechanically connected by adherens junctions as demonstrated by the extensive N-cadherin immunolabeling at the cell's periphery seen by confocal microscopy and the finding of corresponding structures in TEM-images. In contrast, connexin−43 as a marker of gap junctions was found only at low abundance among cardiomyocytes in co-culture spheroids in a similar way as previously observed in hiPSC-CM-only microtissues (Beauchamp et al., 2015). Stem cell-derived cardiomyocytes generally show significantly lower connexin expression levels compared to primary cardiomyocytes from neonatal hearts (Marcu et al., 2015). Overall, the ultrastructural features observed in the spheroids presented similarities to human fetal myocardium while the expression pattern of cTnI in cardiomyocytes and alpha-SMA showed an improved maturation when cardiomyocytes and CF were cultured together.

Similar ultrastructural features as we have found here in spontaneously contracting spheroids have also been observed in electrically paced cardiac spheroids made of hiPSC-CMs (LaBarge et al., 2019). Although the model used here shows spontaneous activity over almost the entire culture time, this does not seem to be sufficient for a substantial maturation of the cells. However, a significantly more advanced structural and functional maturation, including discernable M-bands and positive force-frequency relationship, have been observed in a recent study using hydrogel-based tissues made with early-stage hiPSC-CMs subjected to a high-intensity training for several weeks (Ronaldson-Bouchard et al., 2018). These results in a variety of *in vitro* models of 3D-cultured hiPSC-CMs suggest that at least some sort of extensive, high-intensity training of microtissues is essential to improve the maturation stage of the 3D cultured cardiomyocytes. If scaffold-free spheroids or alternatively bigger tissues in the form of bar- or string-like hydrogels provide a suitable model for a particular study also depends on the required read-outs. Indeed, the spheroid model is unlikely to be used in future for detailed studies on muscle physiology and instead has advantages for rapid and semi-automatic production and analysis of 3D-co-cultures for drug testing applications (Zuppinger, 2019).

### Electrical and Contractile Function

The cardiac identity of hiPSC-CMs has been validated many times and most cardiac cytoskeleton proteins, ion channels and features of EC-coupling have been found expressed and being functional in hiPSC-CMs, although at a fetal level of development and sometimes in transient states between ventricular, nodal, and atrial types (Barbuti et al., 2016; Zuppinger et al., 2017; Zhao et al., 2018). We have investigated spontaneous APs in 2D-cultured hiPSC-CMs, and in cardiac spheroids with and without CF. It has been hypothesized that 3D culture induces a more mature electric phenotype, although in those studies additional interventions were performed such as contractility training or different media formulations (Ronaldson-Bouchard et al., 2018; LaBarge et al., 2019; Valls-Margarit et al., 2019). Resting membrane potential in immature cardiomyocytes has been reported around −60 and −90 mV in adult cells (Barbuti et al., 2016; Zhao et al., 2018), which is in agreement with our results in monolayer-cultured hiPSC-CMs. Furthermore, we did not observe negative effects of the addition of CF in co-culture spheroids with a ratio 4:1 of cardiomyocytes to CF, such as irregular patterns of electrical activity. AP kinetics results cannot be compared directly with the results in 2D culture due to methodological differences, and because of the more depolarized state of hiPSC-CMs limiting the role of Na^+^ (Amin et al., 2010). Overall, these results are in agreement with the observation that CF in 3D conditions revert their activated state seen in monolayer culture as there is no evidence of fibrosis-associated changes. However, we did not find an improvement toward a more mature electrophysiological phenotype in hiPSC-CMs in 3D culture with or without CF. Although, the use of the sharp microelectrode recording directly on 3D spheroids provided a unique opportunity to study electrical activity in a native 3D microenvironment, it also placed a technical limitation to clamp the cell properly, which could explain the much depolarized RMP and slow upstroke velocity observed in our study. Since upstroke velocity is mostly governed by fast INa activation, expression of INa in iPSC from cardiac spheroids needs to be confirmed in future studies.

By using computational video analysis, we have investigated baseline beating rate, the response of cardiac spheroids to electrical field pacing, potential effects of CF in multicellular spheroids, and in monolayer cultures. In co-culture spheroids, applying increasing pacing frequencies resulted in decreasing contraction amplitudes without positive staircase effect. In some spheroids a more accentuated alternans-pattern showed at higher pacing rates. These findings may indicate an inefficient coupling between intracellular calcium entry and release in the hiPSC-CMs (Veerman et al., 2015). Cardiac spheroids generally showed a spontaneous beating rate around 2 Hz at 37°C at 10 days and around 1 Hz at 30 days in culture, which is in agreement with published rates among different hiPSC-lines used for the generation of cardiomyocytes, although a substantial variability of this parameter is commonly encountered in proprietary and commercially available hiPSC-CMs (Barbuti et al., 2016; Horváth et al., 2018). The option to use video analysis as a non-invasive measurement allows to measure spheroid activity at different time points up to several weeks. Regarding the slowing of the spontaneous beating rate over time, it is noteworthy that the beating rate of the normal fetal human heart is in the range of 2–3Hz with a tendency to the lower bound as pregnancy progresses (Pildner von Steinburg et al., 2013). Based on this fact, some researchers have concluded that a trend to lower spontaneous beating rate of hiPSC-CMs or human embryonic stem cell-derived cardiomyocytes is a sign of cellular maturation in the individual tissues or cells, although alternative explanations such as changes in gene expression or the accumulation of catabolic products in spheroids over time would need to be tested in future studies. The beating rate was not depending on the size of the spheroids indicating that the geometry of the tissue is not a limiting factor for the contractile function of these cultures. However, the larger spheroids were beating faster and showed a higher amplitude which may suggest a better compaction of the large tissues facilitating concerted action of groups of cardiomyocytes than in smaller and presumably looser aggregates.

Interestingly, opposite results of co-culturing in spheroids and monolayers were found as co-culture spheroids showed a faster spontaneous beating rate, but monolayer co-cultures a slower spontaneous rate. This is in agreement with earlier studies that showed diminished contractile capacity of 2D-cultured, neonatal rat cardiomyocytes exposed to CF-conditioned medium (LaFramboise et al., 2007) and with a study using murine adult cardiac fibroblasts and stem-cell derived cardiomyocytes in 2D-co-culture (Trieschmann et al., 2016). Fibroblast-conditioned medium was reported to stimulate the percentage of spontaneously beating cardiomyocytes with endothelin-1 secretion from fibroblasts being responsible for the observed effect (Suzuki et al., 1997). A comparison between these two culture types, 3D and 2D, is not trivial because they differ in multiple aspects such as the ratio of fibroblasts to cardiomyocytes, which may change over time as CF continue to proliferate in 2D cultures. Furthermore, the state of CF in monolayer culture is predominantly myofibroblastic, as the assessment of alpha-SMA protein and gene expression has shown, which likely affects paracrine signaling (Mayourian et al., 2018). Re-activation of CF in spheroids has previously been demonstrated by adding TGF-beta or adenoviral transfection of constitutively active G-protein signaling, that caused abnormal electrical activity (Kofron et al., 2017). Since CF and cardiomyocytes are directly interacting in the scaffold-free spheroid, multiple forms of cell-cell communication can occur including paracrine and direct electrotonic interactions that have been previously detected for these two myocardial cell types *in vitro* and *in vivo* (Salvarani et al., 2017). Our results show that the addition of 25% CF to the spheroid improves its contraction rate, rather than impeding beating activity or leading to irregular beating patterns. Regarding contractility in terms of absolute numbers of contractile amplitude in 2D-cultured cardiomyocytes versus spheroids, it needs to be taken into account that the hiPSC-CMs on a culture dish or glass surface are mechanically loaded and their motion is partially confined while the cells in the microtissues show a tissue-like cytoarchitecture and variable orientation. Video analysis captures deformation and motion in the spheroid images; however, this method is not capable of measuring volumetric changes.

Several studies using cancer cell lines have found that cells cultured in 3D models are more resistant to anticancer drugs than in 2D cultures (Rimann and Graf-Hausner, 2012; Imamura et al., 2015). We found similar results here by treating 2D and 3D co-cultures with the know cardiotoxic cancer therapy doxorubicin (Doxo) and using electrical pacing at 3 Hz during video recording sessions. In 2D hiPSC-CM cultures, we noticed a high variability of individual cellular damage after Doxo treatment as previously reported in monolayer cultures of adult rat cardiomyocytes (Sawyer et al., 2002). However, video analysis in 2D culture captured selected regions of interest comprising few cells that are differentially affected by the cardiotoxic treatment, while in spheroids, the contractile performance of the spheroid as an entire beating microtissue is assessed.

## Conclusions

In conclusion, we have investigated characteristics of 3D single- and co-culture of hiPSC-CMs and CF in culture up to 1 month. Selected features were compared with 2D cultures of single or co-cultures. We found that CF in 3D culture lost stress fibers and alpha-SMA expression and reverted to a phenotype similar to normal cardiac tissue. Gene expression of selected genes such as alpha-SMA and troponin-T isoforms showed a more tissue-like pattern in co-cultures of hiPSC-CMs and CF than in single cultures. Ultrastructural features and electrophysiology showed characteristics similar to fetal cardiomyocytes. Beating activity of co-culture spheroids was improved and showed no signs of fibrotic changes. We demonstrate that the use of 3D culture of hiPSC-CMs and CF compared to standard 2D-culture is more closely mimicking the native state of the heart, especially by avoiding CF activation and myofibroblast transformation.

## Data Availability Statement

All datasets generated for this study are included in the article/Supplementary Material.

## Author Contributions

CZ, PB, CJ, LO, and CG performed experiments. CZ and PB designed the study and interpreted the data. IA, DS, TS, and CG contributed materials and discussions. CZ wrote the manuscript, which was critically reviewed and improved by CJ, DS, and TS. All authors read and approved the manuscript.

### Conflict of Interest

IA is employed by the company InSphero AG, Switzerland. CJ is CEO of IKELOS GmbH, Switzerland. The remaining authors declare that the research was conducted in the absence of any commercial or financial relationships that could be construed as a potential conflict of interest.

## Abbreviations

2D: two-dimensional
3D: three-dimensional
alpha-SMA: alpha smooth muscle actin (alpha actin 2)
CF: cardiac fibroblasts
DAPI: 4′,6-diamidin-2-phenylindol
DMEM: Dulbecco's modified eagle medium
Doxo: doxorubicin hydrochloride (adriamycin)
ECM: extracellular matrix
FPS: frames per second
HCF: human cardiac fibroblasts
hiPSC-CMs: human induced-pluripotent stem cell-derived cardiomyocytes
OCT: optimal cutting temperature compound
qPCR: quantitative polymerase chain reaction
SDS: sodium dodecyl sulfate
SEM: scanning electron microscopy
TEM: transmission electron microscopy.

## References

[B1] Abbott A. (2003). Cell culture: biology's new dimension. Nature. 424, 870–872. 10.1038/424870a 12931155

[B2] Agarkova I. Auerbach D. Ehler E. Perriard J. C. (2000). A novel marker for vertebrate embryonic heart, the EH-myomesin isoform. J. Biol. Chem. 275, 10256–10264. 10.1074/jbc.275.14.10256 10744711

[B3] Ali S. R. Ranjbarvaziri S. Talkhabi M. Zhao P. Subat A. Hojjat A. (2014). Developmental heterogeneity of cardiac fibroblasts does not predict pathological proliferation and activation. Circ. Res. 115, 625–635. 10.1161/CIRCRESAHA.115.303794 25037571

[B4] Amin A. S. Tan H. L. Wilde A. A. M. (2010). Cardiac ion channels in health and disease. Heart Rhythm. 7, 117–126. 10.1016/j.hrthm.2009.08.005 19875343

[B5] Archer C. R. Sargeant R. Basak J. Pilling J. Barnes J. R. Pointon A. (2018). Characterization and validation of a human 3d cardiac microtissue for the assessment of changes in cardiac pathology. Sci. Rep. 8:10160. 10.1038/s41598-018-28393-y 29976997 PMC6033897

[B6] Babiarz J. E. Ravon M. Sridhar S. Ravindran P. Swanson B. Bitter H.. (2012). Determination of the human cardiomyocyte mRNA and miRNA differentiation network by fine-scale profiling. Stem Cells Dev. 21, 1956–1965. 10.1089/scd.2011.0357 22050602 PMC4048009

[B7] Banerjee I. Fuseler J. W. Price R. L. Borg T. K. Baudino T. A. (2007). Determination of cell types and numbers during cardiac development in the neonatal and adult rat and mouse. Am. J. Physiol. 293, H1883–H1891. 10.1152/ajpheart.00514.2007 17604329

[B8] Barbuti A. Benzoni P. Campostrini G. Dell'Era P. (2016). Human derived cardiomyocytes: a decade of knowledge after the discovery of induced pluripotent stem cells. Dev. Dyn. 245, 1145–1158. 10.1002/dvdy.24455 27599668

[B9] Beauchamp P. Moritz W. Kelm J. M. Ullrich N. D. Agarkova I. Anson B. (2015). Development and characterization of a scaffold-free 3D spheroid model of iPSC-derived human cardiomyocytes. Tissue Eng. Part C Methods. 21, 852–861. 10.1089/ten.tec.2014.0376 25654582

[B10] Bedada F. B. Chan S. S. K. Metzger S. K. Zhang L. Zhang J. Garry D. J.. (2014). Acquisition of a quantitative, stoichiometrically conserved ratiometric marker of maturation status in stem cell-derived cardiac myocytes. Stem Cell Rep. 3, 594–605. 10.1016/j.stemcr.2014.07.012 25358788 PMC4223713

[B11] Bissell M. J. (2017). Goodbye flat biology - time for the 3rd and the 4th dimensions. J. Cell. Sci. 130, 3–5. 10.1242/jcs.200550 28043963

[B12] Bowers S. L. K. Banerjee I. Baudino T. A. (2010). The extracellular matrix: at the center of it all. J. Mol. Cell. Cardiol. 48, 474–482. 10.1016/j.yjmcc.2009.08.024 19729019 PMC2824065

[B13] Chen W. Frangogiannis N. G. (2013). Fibroblasts in post-infarction inflammation and cardiac repair. Biochim. Biophys. Acta. 1833, 945–953. 10.1016/j.bbamcr.2012.08.023 22982064 PMC3541439

[B14] Chiusa M. Hool S. L. Truetsch P. Djafarzadeh S. Jakob S. M. Seifriz F.. (2012). Cancer therapy modulates VEGF signaling and viability in adult rat cardiac microvascular endothelial cells and cardiomyocytes. J. Mol. Cell. Cardiol. 52, 1164–1175. 10.1016/j.yjmcc.2012.01.022 22326847

[B15] Christoffersson J. Meier F. Kempf H. Schwanke K. Coffee M. Beilmann M.. (2018). A cardiac cell outgrowth assay for evaluating drug compounds using a cardiac spheroid-on-a-chip device. Bioengineering. 5:36. 10.3390/bioengineering5020036 29734702 PMC6027518

[B16] Clément S. Hinz B. Dugina V. Gabbiani G. Chaponnier C. (2005). The N-terminal Ac-EEED sequence plays a role in alpha-smooth-muscle actin incorporation into stress fibers. J. Cell Sci.118, 1395–1404. 10.1242/jcs.01732 15769852

[B17] Desroches B. R. Zhang P. Choi B.-R. King M. E. Maldonado A. E. Li W.. (2012). Functional scaffold-free 3-D cardiac microtissues: a novel model for the investigation of heart cells. Am. J. Physiol. 302, H2031–H2042. 10.1152/ajpheart.00743.2011 22427522 PMC3362102

[B18] Dimitrakis P. Romay-Ogando M.-I. Timolati F. Suter T. M. Zuppinger C. (2012). Effects of doxorubicin cancer therapy on autophagy and the ubiquitin-proteasome system in long-term cultured adult rat cardiomyocytes. Cell Tissue Res. 350, 361–372. 10.1007/s00441-012-1475-8 22864983

[B19] Ehler E. Rothen B. M. Hämmerle S. P. Komiyama M. Perriard J. C. (1999). Myofibrillogenesis in the developing chicken heart: assembly of Z-disk, M-line and the thick filaments. J. Cell. Sci. 112(Pt 10), 1529–1539. 10212147 10.1242/jcs.112.10.1529

[B20] Eppenberger-Eberhardt M. Aigner S. Donath M. Y. Kurer V. Walther P. Zuppinger C.. (1997). IGF-I and bFGF differentially influence atrial natriuretic factor and alpha-smooth muscle actin expression in cultured atrial compared to ventricular adult rat cardiomyocytes. J. Mol. Cell. Cardiol. 29, 2027–2039. 10.1006/jmcc.1997.0408 9281436

[B21] Eschenhagen T. Carrier L. (2019). Cardiomyopathy phenotypes in human-induced pluripotent stem cell-derived cardiomyocytes-a systematic review. Pflugers Arch. 471, 755–768. 10.1007/s00424-018-2214-0 30324321 PMC6475632

[B22] Fleischer S. Jahnke H. G. Fritsche E. Girard M. Robitzki A. A. (2019). Comprehensive human stem cell differentiation in a 2D and 3D mode to cardiomyocytes for long-term cultivation and multiparametric monitoring on a multimodal microelectrode array setup. Biosens. Bioelectron. 126, 624–631. 10.1016/j.bios.2018.10.061 30508787

[B23] Foldes G. Mioulane M. Wright J. S. Liu A. Q. Novak P. Merkely B.. (2011). Modulation of human embryonic stem cell-derived cardiomyocyte growth: a testbed for studying human cardiac hypertrophy? J. Mol. Cell. Cardiol. 50, 367–376. 10.1016/j.yjmcc.2010.10.029 21047517 PMC3034871

[B24] Garzoni L. R. Rossi M. I. D. de Barros A. P. D. N. Guarani V. Keramidas M. Balottin L. B. L. (2009). Dissecting coronary angiogenesis: 3D co-culture of cardiomyocytes with endothelial or mesenchymal cells. J. Exp. Cell Res. 315, 3406–3418. 10.1016/j.yexcr.2009.09.016 19769963

[B25] Harder B. A. Hefti M. A. Eppenberger H. M. Schaub M. C. (1998). Differential protein localization in sarcomeric and nonsarcomeric contractile structures of cultured cardiomyocytes. J. Struct. Biol. 122, 162–175. 10.1006/jsbi.1998.3981 9724617

[B26] Hilse K. E. Rupprecht A. Egerbacher M. Bardakji S. Zimmermann L. Wulczyn A. E. M. S.. (2018). The expression of uncoupling protein 3 coincides with the fatty acid oxidation type of metabolism in adult murine heart. Front. Physiol. 9:747. 10.3389/fphys.2018.00747 29988383 PMC6024016

[B27] Horváth A. Lemoine M. D. Löser A. Mannhardt I. Flenner F. Uzun A. U. (2018). Low resting membrane potential and low inward rectifier potassium currents are not inherent features of hiPSC-derived cardiomyocytes. Stem Cell Rep. 10, 822–833. 10.1016/j.stemcr.2018.01.012 PMC5918194 29429959

[B28] Hutson C. B. Nichol J. W. Aubin H. Bae H. Yamanlar S. Al-Haque S.. (2011). Synthesis and characterization of tunable poly(ethylene glycol): gelatin methacrylate composite hydrogels. Tissue Eng. Part A. 17, 1713–1723. 10.1089/ten.tea.2010.0666 21306293 PMC3118706

[B29] Imamura Y. Mukohara T. Shimono Y. Funakoshi Y. Chayahara N. Toyoda M.. (2015). Comparison of 2D- and 3D-culture models as drug-testing platforms in breast cancer. Oncol. Rep. 33, 1837–1843. 10.3892/or.2015.3767 25634491

[B30] Kloss D. Fischer M. Rothermel A. Simon J. C. Robitzki A. A. (2008). Drug testing on 3D in vitro tissues trapped on a microcavity chip. Lab Chip. 8, 879–884. 10.1039/b800394g 18497906

[B31] Kofron C. M. Kim T. Y. King M. E. Xie A. Feng F. Park E. (2017). Gq-activated fibroblasts induce cardiomyocyte action potential prolongation and automaticity in a 3D microtissue environment. Am. J. Physiol. Heart Circ. Physiol. 313, H810–H827. 10.1152/ajpheart.00181.2017 28710068 PMC5668610

[B32] LaBarge W. Mattappally S. Kannappan R. Fast V. G. Pretorius D. Berry J. L. (2019). Maturation of three-dimensional, hiPSC-derived cardiomyocyte spheroids utilizing cyclic, uniaxial stretch and electrical stimulation. PLoS ONE 14:e0219442 10.1371/journal.pone.0219442 31276558 PMC6611624

[B33] LaFramboise W. A. Scalise D. Stoodley P. Graner S. R. Guthrie R. D. Magovern J. A.. (2007). Cardiac fibroblasts influence cardiomyocyte phenotype *in vitro*. Am. J. Physiol. Cell Physiol. 292, C1799–C1808. 10.1152/ajpcell.00166.2006 17229813

[B34] Landry N. M. Cohen S. Dixon I. M. C. (2017). Periostin in cardiovascular disease and development: a tale of two distinct roles. Basic Res. Cardiol. 113, 1. 10.1007/s00395-017-0659-5 29101484

[B35] Lapp H. Bruegmann T. Malan D. Friedrichs S. Kilgus C. Heidsieck A.. (2017). Frequency-dependent drug screening using optogenetic stimulation of human iPSC-derived cardiomyocytes. Sci. Rep. 7:9629. 10.1038/s41598-017-09760-7 28851973 PMC5575076

[B36] Lee M. O. Jung K. B. Jo S. J. Hyun S. A. Moon K. S. Seo J. W.. (2019). Modelling cardiac fibrosis using three-dimensional cardiac microtissues derived from human embryonic stem cells. J. Biol. Eng. 13:15. 10.1186/s13036-019-0139-6 30809271 PMC6375184

[B37] Li Y. Asfour H. Bursac N. (2017). Age-dependent functional crosstalk between cardiac fibroblasts and cardiomyocytes in a 3D engineered cardiac tissue. Acta Biomater. 55, 120–130. 10.1016/j.actbio.2017.04.027 28455218 PMC5509073

[B38] Lim C. C. Zuppinger C. Guo X. Kuster G. M. Helmes M. Eppenberger H. M.. (2004). Anthracyclines induce calpain-dependent titin proteolysis and necrosis in cardiomyocytes. J. Biol. Chem. 279, 8290–8299. 10.1074/jbc.M308033200 14676206

[B39] Mayourian J. Ceholski D. K. Gonzalez D. M. Cashman T. J. Sahoo S. Hajjar R. J.. (2018). Physiologic, pathologic, and therapeutic paracrine modulation of cardiac excitation-contraction coupling. Circ. Res. 122, 167–183. 10.1161/CIRCRESAHA.117.311589 29301848 PMC5886757

[B40] Miragoli M. Gaudesius G. Rohr S. (2006). Electrotonic modulation of cardiac impulse conduction by myofibroblasts. Circ. Res. 98, 801–810. 10.1161/01.RES.0000214537.44195.a3 16484613

[B41] Norris R. A. Moreno-Rodriguez R. Hoffman S. Markwald R. R. (2009). The many facets of the matricelluar protein periostin during cardiac development, remodeling, and pathophysiology. J. Cell Comm. Signal. 3, 275–286. 10.1007/s12079-009-0063-5 19798597 PMC2778583

[B42] Ongstad E. Kohl P. (2016). Fibroblast-myocyte coupling in the heart: potential relevance for therapeutic interventions. J. Mol. Cell. Cardiol. 91, 238–246. 10.1016/j.yjmcc.2016.01.010 26774702 PMC5022561

[B43] Pfaffl M. W. (2001). A new mathematical model for relative quantification in real-time RT-PCR. Nucleic Acids Res. 29, e45–45. 10.1093/nar/29.9.e45 11328886 PMC55695

[B44] Pfannkuche K. Neuss S. Pillekamp F. Frenzel L. P. Attia W. Hannes T.. (2010). Fibroblasts facilitate the engraftment of embryonic stem cell-derived cardiomyocytes on three-dimensional collagen matrices and aggregation in hanging drops. Stem Cells Dev. 19, 1589–1599. 10.1089/scd.2009.0255 20175666

[B45] Pildner von Steinburg S. Boulesteix A. L. Lederer C. Grunow S. Schiermeier S. Hatzmann W. (2013). What is the “normal” fetal heart rate? PeerJ. 1:e82 10.7717/peerj.82 23761161 PMC3678114

[B46] Pöling J. Gajawada P. Lörchner H. Polyakova V. Szibor M. Böttger T.. (2012). The Janus face of OSM-mediated cardiomyocyte dedifferentiation during cardiac repair and disease. Cell Cycle. 11, 439–445. 10.4161/cc.11.3.19024 22262173

[B47] Polonchuk L. Chabria M. Badi L. Hoflack J.-C. Figtree G. Davies M. J.. (2017). Cardiac spheroids as promising *in vitro* models to study the human heart microenvironment. Sci. Rep. 7:7005. 10.1038/s41598-017-06385-8 28765558 PMC5539326

[B48] Quinn T. A. Camelliti P. Rog-Zielinska E. A. Siedlecka U. Poggioli T. O'Toole E. T.. (2016). Electrotonic coupling of excitable and nonexcitable cells in the heart revealed by optogenetics. Proc. Natl. Acad. Sci. U.S.A. 113, 14852–14857. 10.1073/pnas.1611184114 27930302 PMC5187735

[B49] Rimann M. Graf-Hausner U. (2012). Synthetic 3D multicellular systems for drug development. Curr. Opin. Biotechnol. 23, 803–809. 10.1016/j.copbio.2012.01.011 22326911

[B50] Ronaldson-Bouchard K. Ma S. P. Yeager K. Chen T. Song L. Sirabella D.. (2018). Advanced maturation of human cardiac tissue grown from pluripotent stem cells. Nature 556, 239–243. 10.1038/s41586-018-0016-3 29618819 PMC5895513

[B51] Sadeghi A. H. Shin S. R. Deddens J. C. Fratta G. Mandla S. Yazdi I. K.. (2017). Engineered 3D cardiac fibrotic tissue to study fibrotic remodeling. Adv. Healthc. Mater. 6:1601434. 10.1002/adhm.201601434 28498548 PMC5545804

[B52] Sala L. van Meer B. J. Tertoolen L. T. Bakkers J. Bellin M. Davis R. P.. (2017). MUSCLEMOTION: a versatile open software tool to quantify cardiomyocyte and cardiac muscle contraction *in vitro* and *in vivo*. Circ. Res. 122, e5–e16. 10.1161/CIRCRESAHA.117.312067 29282212 PMC5805275

[B53] Salvarani N. Maguy A. De Simone S. A. Miragoli M. Jousset F. Rohr S. (2017). TGF-β1 (Transforming Growth Factor-β1) plays a pivotal role in cardiac myofibroblast arrhythmogenicity. Circ. Arrhythm. Electrophysiol. 10:e004567. 10.1161/CIRCEP.116.004567 28500173

[B54] Sawyer D. B. Zuppinger C. Miller T. A. Eppenberger H. M. Suter T. M. (2002). Modulation of anthracycline-induced myofibrillar disarray in rat ventricular myocytes by neuregulin-1beta and anti-erbB2: potential mechanism for trastuzumab-induced cardiotoxicity. Circulation 105, 1551–1554. 10.1161/01.CIR.0000013839.41224.1C 11927521

[B55] Schwartz K. Carrier L. Lompr,é A. M. Mercadier J. J. Boheler K. R. (1992). Contractile proteins and sarcoplasmic reticulum calcium-ATPase gene expression in the hypertrophied and failing heart. Basic Res. Cardiol. 87(Suppl 1), 285–290. 10.1007/978-3-642-72474-9_24 1386731

[B56] Serini G. Gabbiani G. (1999). Mechanisms of myofibroblast activity and phenotypic modulation. J. Exp. Cell Res. 250, 273–283. 10.1006/excr.1999.4543 10413583

[B57] Simpson D. G. Terracio L. Terracio M. Price R. L. Turner D. C. Borg T. K. (1994). Modulation of cardiac myocyte phenotype *in vitro* by the composition and orientation of the extracellular matrix. J. Clin. Pharmacol. 161, 89–105. 10.1002/jcp.1041610112 7929612

[B58] Sirenko O. Hancock M. K. Crittenden C. Hammer M. Keating S. Carlson C. B.. (2017). Phenotypic assays for characterizing compound effects on induced pluripotent stem cell-derived cardiac spheroids. ASSAY Drug Dev. Technol. 15, 280–296. 10.1089/adt.2017.792 28837356

[B59] Souders C. A. Bowers S. L. K. Baudino T. A. (2009). Cardiac fibroblast: the renaissance cell. Circ. Res. 105, 1164–1176. 10.1161/CIRCRESAHA.109.209809 19959782 PMC3345531

[B60] Suzuki T. Tsuruda A. Katoh S. Kubodera A. Mitsui Y. (1997). Purification of endothelin from a conditioned medium of cardiac fibroblastic cells using beating rate assay of myocytes cultured in a serum-free medium. J. Mol. Cell. Cardiol. 29, 2087–2093. 10.1006/jmcc.1997.0443 9281441

[B61] Timolati F. Anliker T. Groppalli V. Perriard J.-C. Eppenberger H. M. Suter T. M.. (2009). The role of cell death and myofibrillar damage in contractile dysfunction of long-term cultured adult cardiomyocytes exposed to doxorubicin. J. Mol. Cell. Cardiol. 61, 25–36. 10.1007/s10616-009-9238-4 19890731 PMC2795142

[B62] Timolati F. Timolati F. Ott D. Ott D. Pentassuglia L. Pentassuglia L.. (2006). Neuregulin-1 beta attenuates doxorubicin-induced alterations of excitation–contraction coupling and reduces oxidative stress in adult rat cardiomyocytes. J. Mol. Cell. Cardiol. 41, 845–854. 10.1016/j.yjmcc.2006.08.002 17005195

[B63] Tomasek J. J. Gabbiani G. Hinz B. Chaponnier C. Brown R. A. (2002). Myofibroblasts and mechano-regulation of connective tissue remodelling. Nat. Rev. Mol. Cell Biol. 3, 349–363. 10.1038/nrm809 11988769

[B64] Trieschmann J. Bettin D. Haustein M. Köster A. Molcanyi M. Halbach M.. (2016). The interaction between adult cardiac fibroblasts and embryonic stem cell-derived cardiomyocytes leads to proarrhythmic changes in *in vitro* cocultures. Stem Cells Int. 2016, 2936126–2936112. 10.1155/2016/2936126 26880949 PMC4736407

[B65] Valls-Margarit M. Iglesias-Garcia O. Di Guglielmo C. Sarlabous L. Tadevosyan K. Paoli R.. (2019). Engineered macroscale cardiac constructs elicit human myocardial tissue-like functionality. Stem Cell Rep. 13, 207–220. 10.1016/j.stemcr.2019.05.024 31231023 PMC6626888

[B66] van Meer B. J. Tertoolen L. G. J. Mummery C. L. (2016). Concise review: measuring physiological responses of human pluripotent stem cell derived cardiomyocytes to drugs and disease. Stem Cells. 34, 2008–2015. 10.1002/stem.2403 27250776 PMC5113667

[B67] Veerman C. C. Kosmidis G. Mummery C. L. Casini S. Verkerk A. O. Bellin M. (2015). Immaturity of human stem-cell-derived cardiomyocytes in culture: fatal flaw or soluble problem? Stem Cells Dev. 24, 1035–1052. 10.1089/scd.2014.0533 25583389

[B68] Verjans E.-T. Doijen J. Luyten W. Landuyt B. Schoofs L. (2018). Three-dimensional cell culture models for anticancer drug screening: worth the effort? J. Cell. Physiol. 233, 2993–3003. 10.1002/jcp.26052 28618001

[B69] Zhao Z. Lan H. El-Battrawy I. Li X. Buljubasic F. Sattler K.. (2018). Ion channel expression and characterization in human induced pluripotent stem cell-derived cardiomyocytes. Stem Cells Int. 2018, 6067096–6067014. 10.1155/2018/6067096 29535773 PMC5835237

[B70] Zuppinger C. (2019). 3D cardiac cell culture: a critical review of current technologies and applications. Front. Cardiovasc. Med. 6:87. 10.3389/fcvm.2019.00087 31294032 PMC6606697

[B71] Zuppinger C. Gibbons G. Dutta-Passecker P. Segiser A. Most H. Suter T. M. (2017). Characterization of cytoskeleton features and maturation status of cultured human iPSC-derived cardiomyocytes. Euro. J. Histochem. 61:2763. 10.4081/ejh.2017.2763 28735524 PMC5484009

[B72] Marcu I. C. Illaste A. Heuking P. Jaconi M. E. Ullrich N. D. (2015). Functional characterization and comparison of intercellular communication in stem cell-derived cardiomyocytes. Stem Cells. 33, 2208–2218. 10.1002/stem.2009 25968594

