# Supplementary material for: 3D Co-culture of hiPSC-Derived Cardiomyocytes With Cardiac Fibroblasts Improves Tissue-Like Features of Cardiac Spheroids
Source: Front Mol Biosci. 2020 Feb 14;7:14. doi: 10.3389/fmolb.2020.00014 (PMC7033479; doi:10.3389/fmolb.2020.00014)
Supplement: Supplementary file 6 [file Data_Sheet_1.doc]

**SUPPLEMENTAL MATERIAL**

**Supplemental Methods**

Recording of calcium transients

For the recording of calcium signals shown in supplemental figure 1, spheroids were stained and measured in standard cardiomyocyte culture medium. The calcium-sensitive fluorescent dye Calbryte520-AM (AAT Bioquest, Sunnyvale, USA) was solved as a 5 milliM stock solution in anhydrous DMSO and stored at -20C until use. To avoid motion artefacts, the specific inhibitor of myosin ATPase blebbistatin (Merck) was used at 10 microM in the staining and recording medium. Spheroids were collected in a tube and stained with 5 microM Calbryte520-AM for 1 hour at 37ºC in the dark, then washed with medium without dye for 15 minutes, then transferred to glass-bottom dishes in the warmed chamber on the microscope. Recordings were made using a Nikon inverted microscope, a 40x lens with oil immersion and FITC fluorescence filters. A Nikon DS-Ri CCD camera was used in 4x binning mode to record images of 320x256 pixels at 25 frames per second (fps), and ImageJ software for producing line-profiles of mean pixel intensities of selected regions of interest.

**Supplemental material legends**

**Movie 1** is a composite of two movies showing the same freshly isolated, adult rat ventricular cardiomyocyte. See our previous publication (Timolati et al. 2006) for methods of cell isolation and culture of these cells. Freshly isolated adult cardiomyocytes do not show spontaneous contractions unless induced. Here we made use of electrical field pacing at 1 and 2 Hz for calibrating and validating the computational video analysis software for cardiac spheroids and 2D-cultures.

**Movie 2** is showing phase contrast microscopy images recorded at 37ºC of two beating cardiac spheroids cultured for 10 days, on the top a spheroid without and below with 25% fibroblasts. Shown is first to the left the rate of spontaneous contractions, followed by movies of field-pacing at increasing rates.

**Movie 3** is showing fluorescence microscopy images of a beating cardiac spheroid (co-culture with fibroblasts) stained with the calcium-sensitive dye Calbryte520-AM. The movie first shows spontaneous contractions and then the result of 2Hz electrical field pacing. The fluorescent signals showed a stable pattern of Ca-release and uptake during the entire observation period.

**Movie 4** is showing fluorescence microscopy images of a small cellular aggregate (50±8 µm in diameter) stained with the calcium-sensitive dye Calbryte520-AM. The non-synchronized fluorescent signals from several individual cardiomyocytes illustrate incomplete or absent electrical coupling in those loosely-packed small aggregates in contrast to the larger spheroids (Movie 3) that show synchronized calcium signals and a more compact structure as demonstrated by the histology data.

**Supplemental figure 1.** A: Still frame of movie 3 showing fluorescent signals from the calcium-sensitive dye Calbryte520-AM from a co-culture spheroid. The superimposed white rectangle in the center of the cross hairs shows the region of interest that was chosen for further analysis. B: X-T representation of the intensity of fluorescent signals in the selected region over the full duration of the recording which had a total length of 26 seconds. C: Line-profile of the mean pixel intensity over time shown in frames (at 25 fps sampling) demonstrating the kinetics of calcium transients and the regular pattern of calcium-cycling.
