# Supplementary figures and images for: 3D Co-culture of hiPSC-Derived Cardiomyocytes With Cardiac Fibroblasts Improves Tissue-Like Features of Cardiac Spheroids
Source: Front Mol Biosci. 2020 Feb 14;7:14. doi: 10.3389/fmolb.2020.00014 (PMC7033479; doi:10.3389/fmolb.2020.00014)

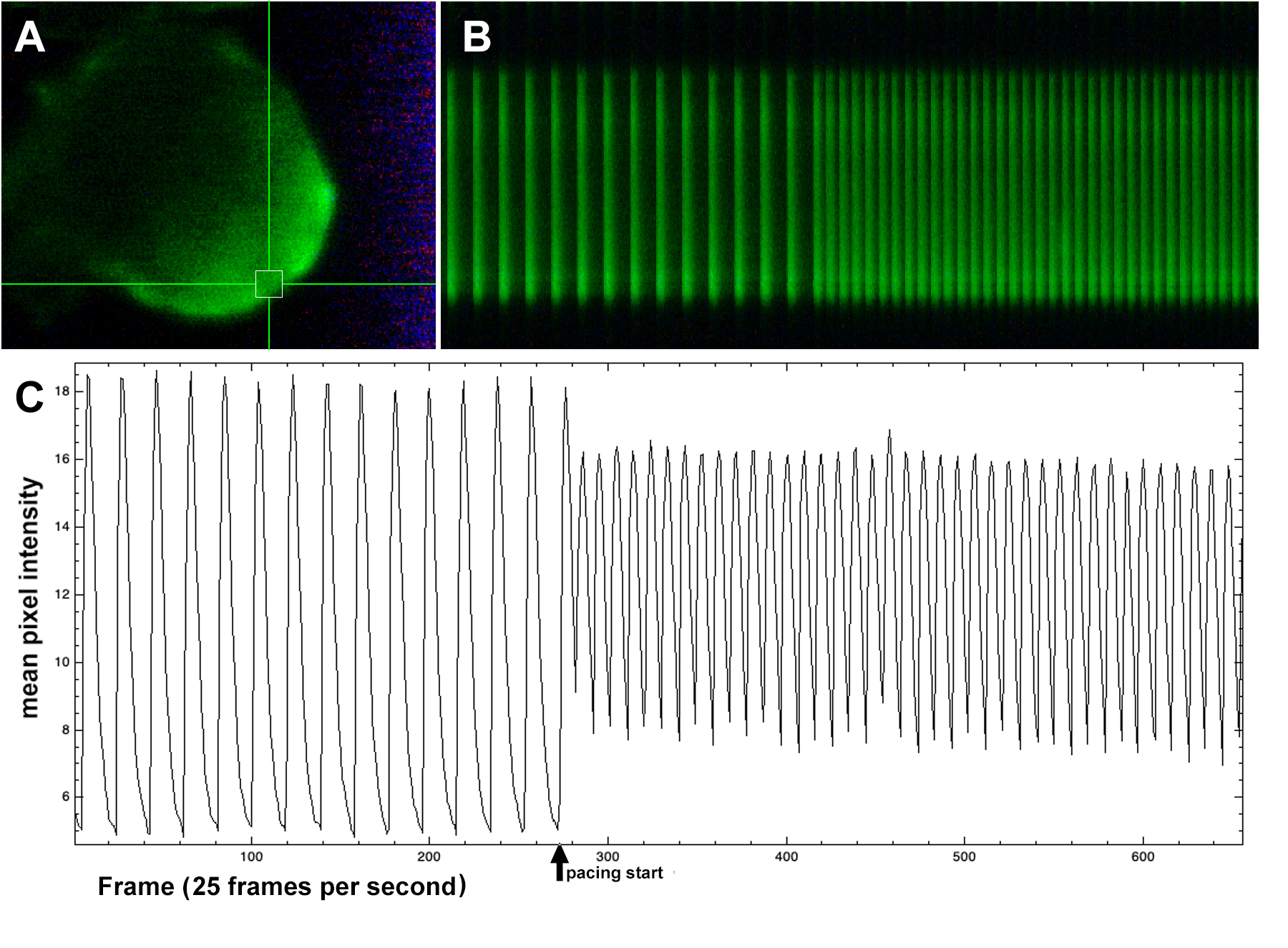

Supplement: Supplementary file 7 [file Image_1.jpg]
